# Sentrin/SUMO Specific Proteases as Novel Tissue-Selective Modulators of Vitamin D Receptor-Mediated Signaling

**DOI:** 10.1371/journal.pone.0089506

**Published:** 2014-02-20

**Authors:** Wai-Ping Lee, Sarita Jena, Declan Doherty, Jaganathan Ventakesh, Joachim Schimdt, Julie Furmick, Tim Widener, Jana Lemau, Peter W. Jurutka, Paul D. Thompson

**Affiliations:** 1 School of Biomedical Sciences, University of Ulster, Coleraine, Northern Ireland, United Kingdom; 2 Divison of Mathematical and Natural Sciences, Arizona State University at the West Campus, Glendale, Arizona, United States of America; Nihon University School of Medicine, Japan

## Abstract

Vitamin D receptor (VDR) is a substrate for modification with small ubiquitin-like modifier (SUMO). To further assess the role of reversible SUMOylation within the vitamin D hormonal response, we evaluated the effects of sentrin/SUMO-specific proteases (SENPs) that can function to remove small ubiquitin-like modifier (SUMO) from target proteins upon the activities of VDR and related receptors. We report that SENP1 and SENP2 strikingly potentiate ligand-mediated transactivation of VDR and also its heterodimeric partner, retinoid X receptor (RXRα) with depletion of cellular SENP1 significantly diminishing the hormonal responsiveness of the endogenous vitamin D target gene *CYP24A1*. We find that SENP-directed modulation of VDR activity is cell line-dependent, achieving potent modulatory effects in Caco-2 and HEK-293 cells, while in MCF-7 cells the vitamin D signal is unaffected by any tested SENP. In support of their function as novel modulators of the vitamin D hormonal pathway we demonstrate that both SENP1 and SENP2 can interact with VDR and reverse its modification with SUMO2. In a preliminary analysis we identify lysine 91, a residue known to be critical for formation and DNA binding of the VDR-RXR heterodimer, as a minor SUMO acceptor site within VDR. In combination, our results support a repressor function for SUMOylation of VDR and reveal SENPs as a novel class of VDR/RXR co-regulatory protein that significantly modulate the vitamin D response and which could also have important impact upon the functionality of both RXR-containing homo and heterodimers.

## Introduction

The small ubiquitin-related modifier (SUMO) represents a novel class of ubiquitin-like protein that has emerged over the past decade as a key regulator of cellular protein activity [1]. In contrast to ubiquitination which results primarily in substrate degradation, conjugation with SUMO can alter the properties of the target protein in a variety of ways, including its profile of localization, interactions, and susceptibility to alternate modification processes [2]. The current list of known SUMOylated substrates is represented primarily (although not exclusively) by nuclear proteins, the modifications of which establish SUMOylation as a process integral to gene expression, nuclear body formation and the maintenance of genomic/chromosomal stability. In addition, there is accumulating evidence that link SUMO-related events to certain cancers as well as neurodegenerative disorders such as Alzheimer’s and Parkinson’s disease [3]–[6]. As common to many regulatory mechanisms, the conjugation of a target substrate with SUMO can be reversed, a process facilitated through the activities of a family of sentrin/SUMO-specific proteases (SENPs) [7]. While the human genome is thought to encode approximately 100 enzymes of a putative deubiquitination function [8], there are in contrast only six known members of the mammalian SENP family that differ from each other through their patterns of expression, subcellular localization, SUMO paralog specificity, and predominant reaction catalyzed among the events of SUMO processing, deconjugation or chain-editing [9], [10]. Although it remains to be determined as to how a large number of SUMOylated proteins may be selectively processed through a comparatively limited number of SENPs, it would appear that these proteases do not exhibit redundant or overlapping enzymatic activities but rather exert their biological effects in a precise manner [11].

Several members of the superfamily of nuclear hormone receptors (NHRs), including the steroid receptors for androgen (AR), estrogen (ER) in addition to ‘metabolic-sensors’ such as liver X receptor α (LXRα), pregnane X receptor (PXR) and peroxisome proliferator-activated receptor γ (PPARγ) are known to be directly SUMOylated with this modification having significant impact upon their function as transcriptional activators or transrepressors [12]–[17]. NHR-directed regulation of gene expression involves their recruitment of protein metacomplexes to the target gene promoter that dictate chromatin accessibility and serve as molecular bridges with the basal transcriptional machinery. As a number of receptor interacting proteins within these complexes, such as steroid receptor coactivator-1 (SRC-1), glucocortocoids receptor-interacting protein-1 (GRIP1), and histone deacetylase-1 (HDAC1), are known to be SUMOylated [18]–[20], it is clear that conjugation with SUMO and its subsequent reversal through SENP activity can potentially modulate NHR-mediated signaling at several key regulatory points and may represent a means for achieving subtle differential cell and gene specific responses to receptor stimuli.

We recently reported vitamin D receptor (VDR) to be a SUMOylated protein, a process enhanced through interaction with protein inhibitor activated STAT 4 (PIAS4) which also serves as a potent inhibitor of the transcriptional response to 1,25(OH)_2_D_3_ (1,25D) [21]. As our data associate VDR SUMOylation with repressed level of receptor transactivation, we employed a series of functional assays to assess the impact of SENP co-expression upon the activities of VDR in relation to effects achieved with other members of the heterodimerizing class of receptor. Our data reveal that SENP1 and SENP2 have the capacity to directly associate with VDR and serve as novel cell-type and potentially gene-specific modulators of the 1,25D response. Intriguingly both SENP1 and SENP2 facilitate the removal of SUMO2 from modified VDR. Our data implicate that SUMOylation of VDR and its reversal through SENP-mediated activity may represent a means to achieve ‘fine-tuning’ of the cellular responses to dietary and endocrine-derived ligands for VDR.

## Materials and Methods

### Cell Culture and Ligands

HEK-293, CHO-K1 and MCF-7 cells were obtained from the European Collection of Cell Culture (ECACC). Caco-2 cells were purchased from American Tissue Culture Collection (ATCC). All cells were cultured in a 37°C incubator with 5% CO_2_ with all media and supplements obtained from GIBCO (Invitrogen, Carlsbad, CA), unless otherwise stated. The standard culturing conditions for each cell line and passage number when used were: HEK-293 (p71–82) Dulbecco’s modified eagle medium (DMEM) +10% fetal bovine serum (FBS), 2 mM L-glutamine, 50 units/ml penicillin G and 50 µg/ml streptomycin; CHO KI (p15–20) DMEM-F12+10% FBS, 2 mM L-glutamine, 50 units/ml penicillin G and 50 mg/ml streptomycin; MCF-7 (p22–26) DMEM +10% FBS, 1% L-Glutamine, and 1% non-essential amino acids; Caco-2 (p22–29) DMEM containing high glucose (Sigma, St. Louis, MO) +15% FBS.

The ligands for VDR (1,25(OH)_2_D_3_), RXRα (9-*cis* retinoic acid), LXRα (TO901317) and PPARγ (GW1929) were all purchased from Sigma. The FXR ligand GW474066 was a generous gift of Dr. Stacey Jones, Glaxosmithkline, Research Triangle Park, North Carolina.

### Plasmids

Expression plasmids for full length human VDR and RXRα (pSG5hVDR and pSG5hRXRα, respectively) were kindly provided by Prof Mark Haussler, University of Arizona, College of Medicine, Phoenix. The construct pcDNAV5-VDR that encodes human VDR ‘tagged’ with the V5 epitope has been previously described [21]. Expression vectors for human SENP1 (pFLAG-CMV-SENP1) and SENP2 (pFLAG-CMV-SENP2) were a generous gift from Prof. Edward T. H. Yeh of University of Texas M. D. Anderson Cancer Center. ‘Entry’ clones encoding full length human VDR and RXRα, in addition to the ligand binding domains (LBD) of human VDR (aa96–427), hRXRα (aa197–462), LXRα (aa104–447) FXR (aa156–472), and PPARγ (aa166–477) were initially generated via PCR from a human fetal brain cDNA library, followed by insertion of each purified DNA fragment into the pDONR201 vector via ‘BP clonase’ reaction (Invitrogen). Entry clones for SENP1 and 2 were similarly produced using products amplified from their respective expression vectors as template. Gal4-based hybrid expression vectors for each nuclear receptor and SENP evaluated were then obtained through the ‘LR clonase’ reaction using the appropriate entry clone combined with either of the mammalian hybrid expression vectors pCMVBD or pCMVAD (Stratagene, La Jolla, CA), previously modified to be gateway compatible. All constructs were subjected to DNA sequencing to confirm correct identity and reading frame for each gene insert. Vectors expressing Ubc9 and His-tagged SUMO2 have been previously described [21]. The firefly luciferase-based reporter construct pMCS-24OHase contained a 5500 bp fragment of the promoter region from the human vitamin D_3_ 24 hydroxylase (*CYP24A1*) gene. For experiments based upon the activities of Gal4DBD-nuclear receptor hybrid proteins, the transcriptional responses to cognate ligand were monitored through the luciferase signal generated from the pFLUC reporter (Stratagene) that contains five copies of the Gal4 response element.

### Site-Directed Mutagenesis

Synthesis of point mutations within the pcDNAV5-VDR and Gal4-based expression constructs for VDR was accomplished through the Quikchange XL site-directed mutagenesis system (Agilent Technologies) using the following mutagenic primer pairs;

K91RVDR.

5′-CATCGGCATGATGAGGGAGTTCATTCTGAC-3′

5′-GTCAGAATGAACTCCCTCATCATGCCGATG-3′

K103RVDR.

5′-GAAGTGCAGAGGAGGCGGGAGATGATCC-3′

5′-GGATCATCTCCCGCCTCCTCTGCACTTC-3′

K111RVDR.

5′-GATCCTGAAGCGGAGGGAGGAGGAGGCC-3′

5′-GGCCTCCTCCTCCCTCCGCTTCAGGATC-3′

K399RVDR.

5′-CAATGAGGAGCACTCCAGACAGTACCGCTGCCTCTC-3′

5′-GAGAGGCAGCGGTACTGTCTGGAGTGCTCCTCATTG-3′

K413RVDR.

5′-GAGTGCAGCATGAGGCTAACGCCCCTTG-3′

5′-CAAGGGGCGTTAGCCTCATGCTGCACTC-3′

### Transcriptional Activation Assays

Cultured cells were trypsinized at ∼80% confluency and then seeded unto a 96-well plate at 1×10^4^ cells/well (for CHO-K1) or a 24-well plate at 2–3×10^5^ cells/well (for all other cell types). All cells were seeded in their standard culture media with the exception of MCF-7 cells where phenol red free DMEM supplemented with 5% charcoal stripped FBS was used (plus L-glutamine and NEAAs). After an incubation period of 24 hours, cells were subsequently transfected with the appropriate combinations of plasmid via Lipofectamine 2000 following a protocol based on manufacturer’s instructions. Reporter activity was measured following lysis of cells in passive lysis buffer (Promega) and recording of chemiluminescent signal through use of the Dual-Glo Luciferase Reporter Assay System (Promega). Transfection data was normalized relative to the luciferase signal produced from the constitutively active renilla vector (pRL-TK) and expressed as a mean of relative light units from triplicate assays ± the standard deviations.

### Protein-protein Interaction Experiments

Association between SENP and VDR or RXRα proteins were assessed through both mammalian two hybrid and GST-pulldown methodologies. Two hybrid experiments were performed using CHO-K1 cells under standard culturing conditions. Co-transfection of CHO-K1 cells with the appropriate bait (SENP) and prey (receptor) fusion constructs, in combination with the reporter pFLUC and pRL-TK internal control, were performed using Lipofectamine 2000 based protocol and reporter activity measured using the Dual-Glo luciferase system as described above. pDEST17 plasmids containing cDNA inserts for either SENP1 or 2 were produced through LR clonase reaction and together with the pSG5hRXRα expression construct, were used as templates in an *in vitro* transcription/translation (IVTT) reaction (Promega Corp) to generate [^35^S]-methionine-labeled SENP and hRXRα proteins. GST-hVDR fusion protein, or GST alone, bound to glutathione-coated Sepharose beads were generated as previously described [22]. All beads were then pre-incubated with 10^−6^ M 1,25D or ethanol vehicle for 1 h at 22°C, followed by incubation with 20 µl of the appropriate radiolabeled IVTT lysate for 1 h at 4°C. The beads were then washed extensively as detailed by Jurutka and co-workers [22] and the amount of co-precipitated SENPs or hRXRα detected by electrophoresis of denatured bead samples followed by autoradiography.

### Western Blotting

Whole cell extracts from treated cells was achieved using a RIPA buffer (50 mM Tris HCl pH7.5, 150 mM NaCl, 0.5% IGEPAL, 5 mM EDTA pH8.0 and 10% glycerol). Samples were added to a 4X loading buffer (200 mmol/L Tris HCl pH 6.8, 400 mmol/L β-mercaptoethanol, 8% SDS, 0.4% bromophenol blue and 40% glycerol), heated at 95°C for 5 min and 50 µg of each lysate then fractionated through SDS-PAGE electrophoresis on a 4–12% NuPAGE Bis/Tris gel (Invitrogen). After transfer onto an Immobilon-P membrane (Millipore Corp, Billerica, MA), target or ‘tagged’ proteins were detected using the following antibodies: rat monoclonal (9A7) anti-human VDR (Enzo Life Sciences) at a 1∶5000 dilution; rabbit polyclonal anti-Gal4 DBD antibody (Santa Cruz Biotechnology, Santa Cruz, CA) at a 1∶1000 dilution; mouse monoclonal anti-V5 (Invitrogen) at a 1∶5000 dilution; mouse, mouse monoclonal anti-6xHis (Abcam) at 1∶1000 dilution; mouse monclonal anti-Flag (Sigma) at a 1∶1000 and mouse monoclonal anti β-actin (Sigma) at 1∶10000 dilution. Proteins were visualized using Supersignal West Pico Chemiluminescent solution (Thermo Scientific) and development on autoradiographic film, on a Kodak X-Omat 1000 processor. Following stripping and blocking, all membranes were re-probed with a mouse monoclonal anti-β-actin antibody (Sigma) at 1∶10,000 dilution. The secondary antibody used was a rabbit anti-mouse IgG (whole molecule) peroxidase conjugate antibody (Sigma) and the membrane was processed and developed as described above.

### mRNA Expression Analysis through PCR, qRT-PCR and siRNA Approaches

For studies of the effects of SENP1 over-expression upon endogenous *CYP24A1* gene transcription, Caco-2 cells were plated at 1×10^6^ cells/60 mm plate and co-transfected with pSG5hVDR in combination with the expression construct for SENP1 or equivalent amount of empty parent vector. Cells were treated post-transfection with 10^−8^ M 1,25D at defined time points followed by isolation of total RNA using an Aurum Total RNA Mini Kit (Bio-Rad, Hercules, CA). DNase treated RNA (2 µg) was then reverse transcribed using the iScript cDNA Synthesis Kit (Bio-Rad) and the resultant cDNA employed in PCR reactions containing 10 µL iQ SYBR Green Supermix (Bio-Rad), 1 µL primers, 2 µL of cDNA template sample, and molecular grade water to a final reaction volume of 20µl. Real-time PCR was performed on the human *CYP24A1* gene using 5′-CAGCGAACTGAACAAATGGTCG-3′ and 5′-TCTCTTCTCATACAACACGA-GGCAG-3′ primers (58 bp product). Reactions were performed in 96-well PCR plates and read on a Bio-Rad iCycler iQ Real-Time PCR detection system or an ABI 7500 Fast instrument. Data were analyzed using the comparative Ct method as a means of relative quantitation, normalized to an endogenous reference (*GAPDH* cDNA) and relative to a calibrator (normalized Ct value obtained from vehicle-treated cells) and expressed as 2−ΔΔCt according to Applied Biosystems User Bulletin 2: Rev B, “Relative Quantitation of Gene Expression.”

To evaluate how depletion of endogenous SENP1 may impact upon expression of the human *CYP24A1* and *TRPV6* genes, Caco-2 cells were seeded in 6-well plates at 8×10^4^ cells/well and transfected using DharmaFECT1 (Thermo Scientific) following manufacturer’s instructions with siRNA specific for *SENP1* (ON-TARGETplus SMARTpool L-006357-00-0005), or an non-targeting siRNA pool (D-001810-10-05). After incubation in transfection mix for 48 hours, cells were allowed to recover in fresh media before addition of media supplemented with 10^−8^ M 1,25D or vehicle control for a period of 24 hours. Following ligand/vehicle treatments, cells were then harvested and total RNA and protein extracted for analysis. cDNA was generated as described above and PCR performed using the following gene specific primers;

*CYP24A1* (381 bp product).

5′-CTACCGCAAAGAAGGCTACG-3′

5′-TTGGTGTTGAGGCTCTTGTG-3′

*HPRT* (350 bp product).

5′-GACCAGTCAACAGGGGACAT-3′

5′-TAGCTTGCGACCTTGACCAT-3′

*SENP1* (321 bp product).

5′-GGCTGGTTATCAGGCAGTG-3′

5′-CGGAAGTATGGCATGTGTTG-3′.

The resulting PCR products were visualized through electrophoresis using a 1% agarose gel containing 0.2% ethidium bromide. Analysis of expression through real-time PCR approaches for cDNA obtained from siRNA treated samples was accomplished on a Roche Light Cycler 480 using Real Time assay probes specific for human *CYP24A1* (assay ID: 114955), *TRPV6* (assay ID: 110452), *SENP1* (assay ID: 108243) with *HPRT* (assay ID: 102079) representing the endogenous reference gene. Data was analyzed using Advanced Relative Quantification Software (Roche). Extracted protein samples from each treatment group were subjected to immunoblotting as described above.

### Cell-based SUMOylation Assays

Detection of SUMO-modified VDR was performed as previously described [21]. Briefly, HEK293 cells were seeded in 60 mm dishes and transfected with the appropriate construct expressing V5-VDR (2µg), His-SUMO2 (2µg), UBC9 (1µg) Flag-SENP1 or 2 (1µg) or parent vector control. At 48 hours post transfection, cells were harvested and the resulting cell pellets resuspended in ice-cold RIPA buffer and subject to sonication. 20µl of V5 agarose beads (Abcam) was added to 1 mg of the resulting cleared cell lysate diluted with SUMO-IP buffer. After overnight incubation at 4°C and washing (thrice) with SUMO-IP buffer, samples were then resuspended in 3X reducing agent/LDS sampling buffer. After heating at 100°C for 5 minutes followed by centrifugation, the eluted proteins were then analyzed through western blotting.

## Results

### SENPs Interact with VDR and RXRα and Enhance their Ligand-induced Activation

**Figure 1:**
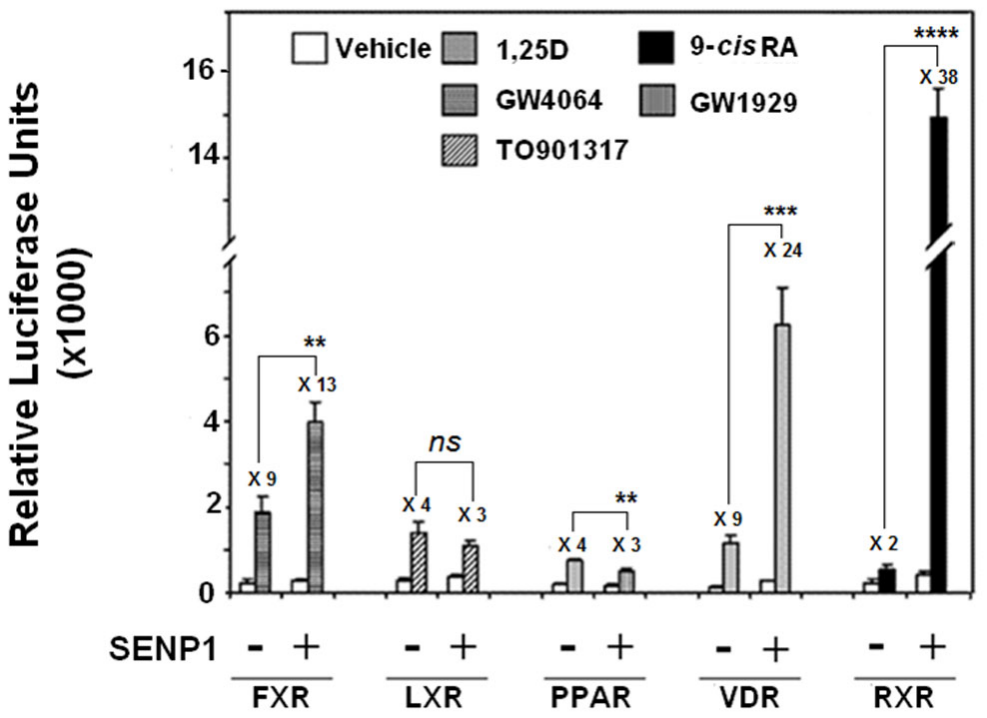
SENP1 selectively potentiates the transcriptional activities of VDR and RXRα. HEK-293 cells were transfected with the pFLUC reporter vector in combination with the appropriate pCMVBD-based expression vector for each Gal4-nuclear receptor (LBD) hybrid protein under evaluation. Where indicated, cells also received the SENP1 expression plasmid, pFLAG-CMV-SENP1 (200 ng), or an equivalent amount of parent vector (minus SENP1 insert) as control. The total amount of DNA in each transfection was kept at a constant value through inclusion of the appropriate amount of empty expression vector. Treated cells were dosed with the appropriate cognate ligand or vehicle control for a period of 24 hours before measurement of luciferase activity. All ligands were used at a concentration of 10^−6 ^M, except 1,25D (10^−8 ^M). After normalization for transfection efficiency based on the activity of the pRL-TK control, results were expressed as relative luciferase units per well. The fold-stimulation (ratio of activity in the presence:absence of ligand) is indicated above each set of bars. Data represents analysis of at least three independent experiments with each treatment run in triplicate (*n = *3) mean ± SD; where *ns* = p≥0.05, ** p = 0.001–0.01, *** p = 0.0001–0.001 and **** p<0.0001.

**Figure 2:**
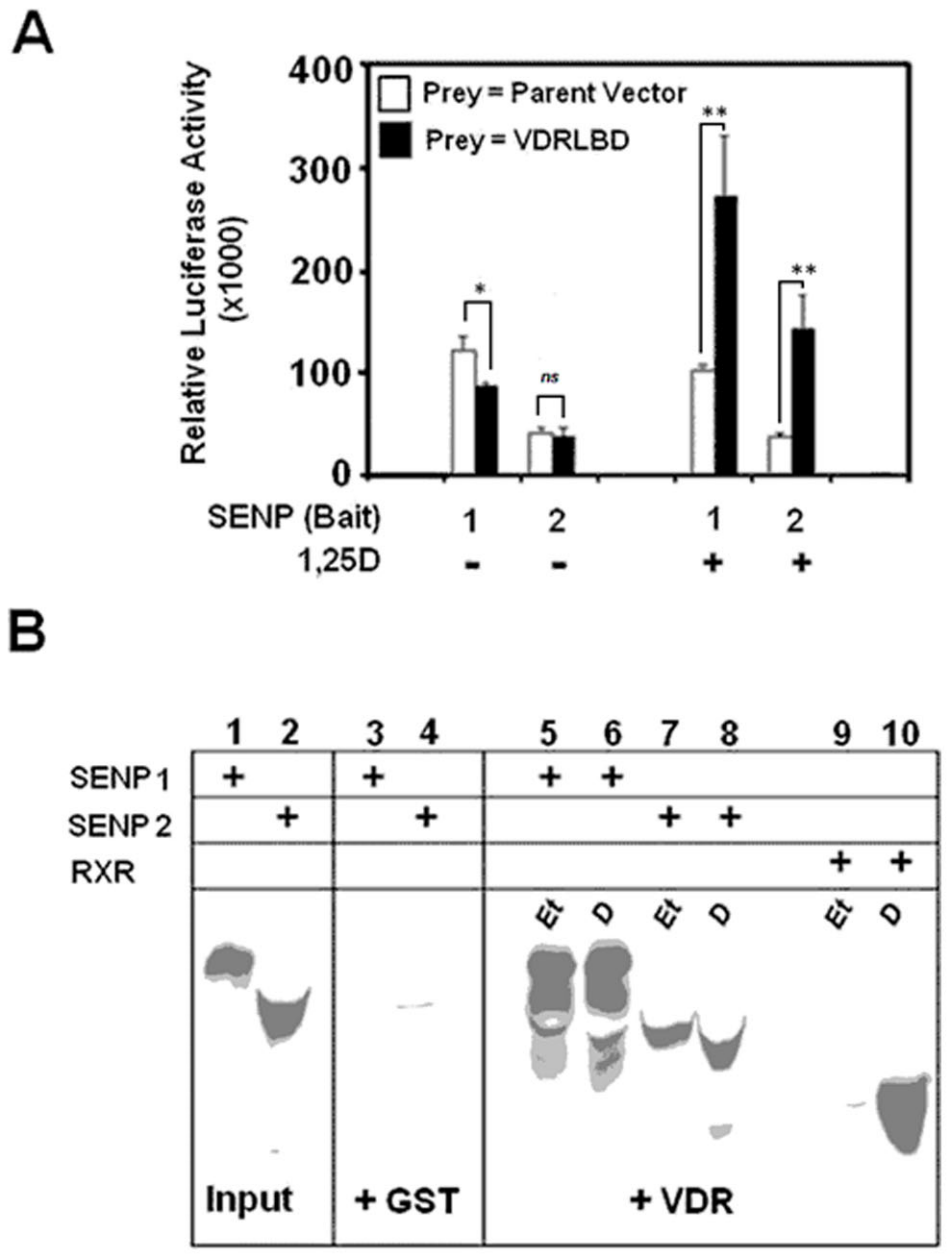
Members of the SENP family directly interact with VDR. **A**. Mammalian two-hybrid assay was employed to assess the abilities of VDR to directly associate with SENP 1 or 2. CHO KI cells were co-transfected with the pFR-LUC reporter along with the indicated combination of bait (pCMVBD-SENP1 or 2) and prey (pCMVAD, pCMVAD-VDRLBD) constructs. Cells were incubated with 1,25D (10^−8 ^M), or vehicle control for a period of 24 hours before measurement of luciferase activity. After normalization for transfection efficiency based on the activity of the pRL-TK control, results were expressed as relative luciferase units per well. Data represents the average of three independent experiments run in triplicate, mean ± S.D; where *ns* = <0.05, * p = 0.01–0.05, ** p = 0.001–0.01. **B**. Interaction between VDR and SENP1 & 2 was monitored through GST-pulldown assay. GST-hVDR fusion protein, or GST alone, bound to glutathione-coated Sepharose beads were pre-incubated with 10^−6^ M 1,25D (D lanes) or ethanol vehicle (Et lanes) for 1 h at 22°C, followed by incubation with 20 µl of [^35^S]-methionine-labeled SENP or RXRα proteins for 1 hour at 4°C. After washing, coprecipitated SENPs or RXRα was detected by electrophoresis of denatured bead samples followed by autoradiography. Aliquots (5%) of all radiolabeled protein are shown in the input lanes (1 & 2) to assess the level of SENP synthesized in the IVTT reaction. Lanes 9 & 10 detail positive controls employing RXRα, an established VDR-interacting protein. Data is representative of three independent experiments.

In order to ascertain if reversible SUMOylation is an integral process to their mechanisms of transactivation, we subjected a select group of nuclear receptors to a functional screen in which we assessed the impact of SENP1 co-expression upon their ligand-induced activation. The nuclear receptors under evaluation (VDR, RXRα, PPARγ, LXRα and FXR) were represented by hybrid constructs consisting of their respective ligand binding domain (LBD) fused to the Gal4 DNA binding domain (DBD). Fig. 1A reveals that SENP1 elicits the most striking effects upon the liganded activities of the VDR and RXRα constructs and increased their transcriptional responses to cognate ligand approximately 3 and 19-fold respectively. In contrast SENP1 co-expression appears to have little impact upon the transactivations of LXRα and PPARγ by their respective cognate ligands above that obtained with the empty vector control. It is noted that both the basal and liganded activities of FXR are increased through the presence of SENP1 which although results in a more modest overall fold increase (X 1.4) in transactivation to those observed for VDR and RXRα, does suggest that this receptor may be subject to modulation through SUMO-related events. In focusing upon their potential role as regulators of the vitamin D pathway, we assessed if SENP1 and also SENP2 can directly interact with VDR. Fig. 2A depicts a mammalian two hybrid experiment in which ‘prey’ constructs encoding the LBD of VDR were paired with ‘bait’ representing SENP1 or SENP2. The data reveal that VDR can associate with the tested SENPs in a manner that would appear to be dependent upon the presence of ligand. Based upon the absolute values of the luciferase signal generated, VDR exhibited highest affinity for SENP1, although the relative fold increase in reporter activity that resulted from the association with receptor was comparable for both SENP isoforms.

We also utilized a ‘pull-down’ methodology to determine if IVTT generated SENP1 and SENP2 have the capacity to interact with a GST-hVDR fusion protein. Fig. 2B indicates that while radiolabeled SENP proteins exhibit no association with the GST control (lanes 3 & 4), both SENP1 (lanes 5 & 6) and SENP2 (lanes 7 & 8) are able to interact with VDR. In contrast to data obtained in Figs. 2A and to the clear hormone-dependent nature of the interaction exhibited between VDR and its RXRα binding partner employed as a positive control (compare lanes 9 & 10) we observe that in the context of the *in vitro* assay, associations between VDR and SENP1 (lanes 5 & 6) and SENP2 (lanes 7 & 8) appear to occur with equal intensity in both vehicle control and 1,25D treated samples.

### SENPs Regulate the Activities of VDR in a Cell-specific Fashion

**Figure 3:**
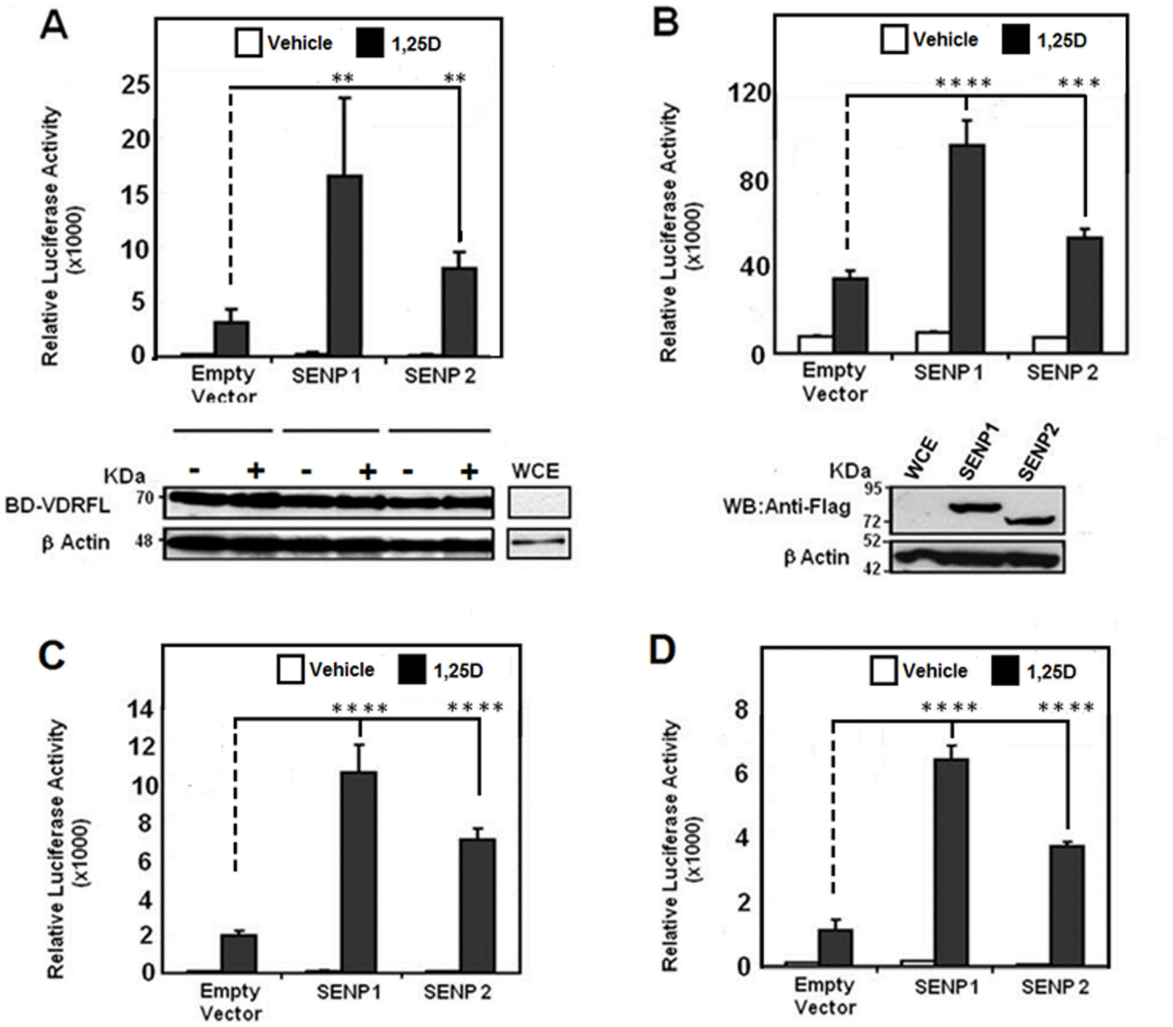
SENPs potentiate transactivation of the full length VDR protein. **A**. HEK-293 cells received pCMVBD-VDRFL that encodes Gal4DBD fused to full length human VDR, in combination with the pFR-LUC reporter and the indicated SENP expression construct or parent vector control. The lower panel depicts an immunoblot analysis in which combined cellular lysates from each treatment group were probed with the antibodies specific for VDR (9A7) and β-actin. **B**. pSG5-hVDR that expresses full length human VDR were co-transfected into HEK-293 cells in combination with the reporter construct pMCS-24OHase, pSG5-hRXRα and the appropriate Flag-SENP expression plasmid or parent vector control. Transfected cells were incubated with 1,25D (10^−8 ^M) or vehicle for 24 hours before measurement of luciferase activity. The lower panel depicts immunoblot analysis of cell lysates transfected with the pFlag-SENP1 or pFlag-SENP2 expression constructs and then probed with the Flag or β-actin specific antibodies, with WCE representing the untransfected whole cell lysate control. The fold stimulation are expressed as means (± SD) and results presented are the average of three independent experiments, where *n = 3* in each assay. **C**. Caco-2 cells were co-transfected with pCMVBD-VDRFL and pFR-LUC reporter in combination with the appropriate SENP expression constructs or parent vector control. **D**. Caco-2 cells received pSG5-hVDR+pSG5-hRXRα, the pMCS-24OHase reporter, together with the indicated SENP expression plasmid or parent vector control. Transfected cells were then incubated with 1,25D (10^−8 ^M) or vehicle control for 24 hours before measurement of luciferase activity. All depicted data represents an average of four independent experiments with values expressed as means (± SD) of triplicate assays (*n* = 3) where ** p = 0.001–0.01, *** p = 0.0001 - 0.001, **** p<0.0001.

**Figure 4:**
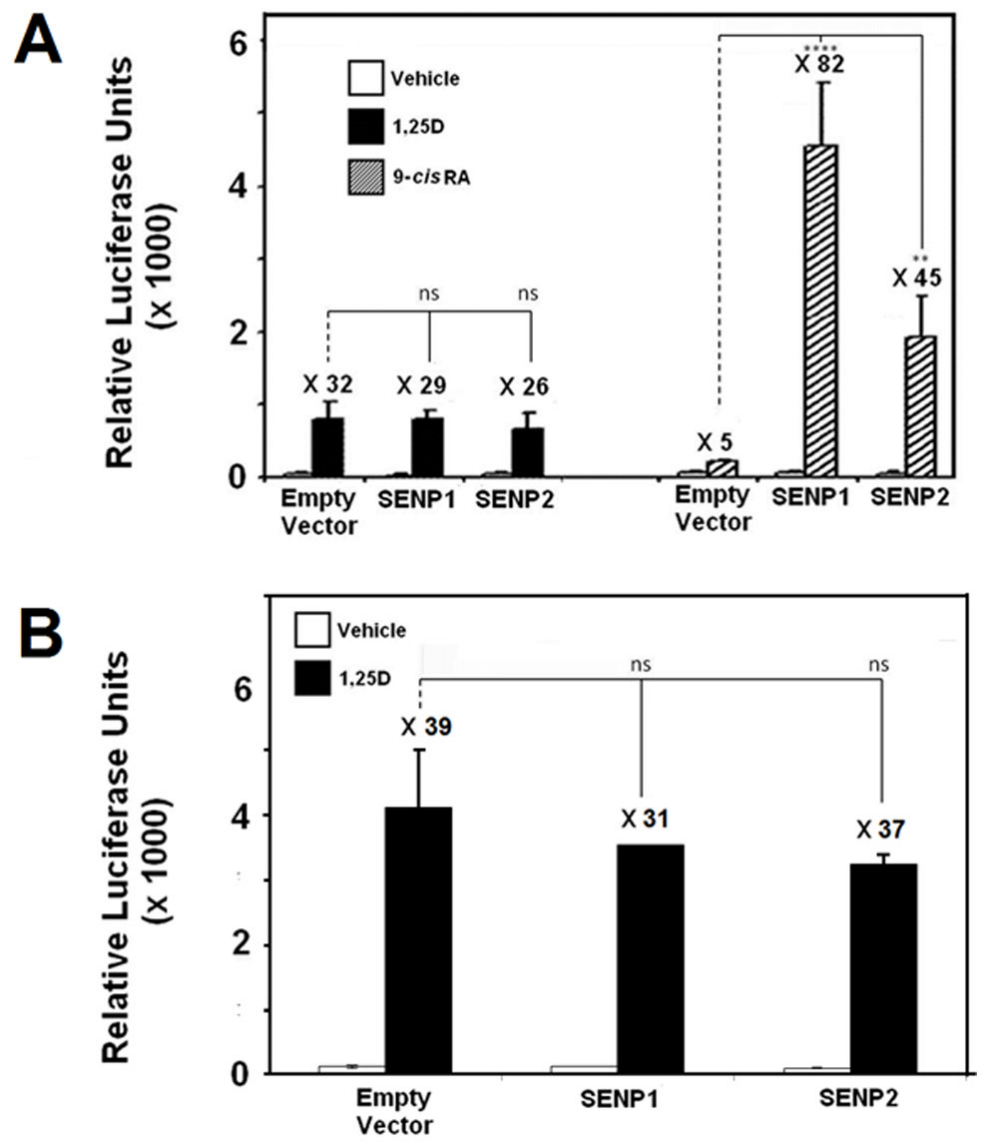
Transcriptional activities of VDR and RXRα in MCF-7 cells are differentially modulated by SENPs. **A**. MCF-7 cells were seeded under conditions defined in methods and received the indicated SENP expression construct or corresponding parent vector control in combination with the pFLUC reporter and pCMVBD-VDRFL or pCMVBD- RXRαFL. **B**. pSG5-hVDR and pSG5-hRXRα were co-transfected into MCF-7 cells in combination with the pMCS-24OHase reporter and appropriate Flag-SENP expression plasmid or parent vector control. Cells were then dosed for 24 hours with the 1,25D (10^−8 ^M) or 9-*cis* RA (10^−6 ^M) cognate ligands or vehicle control where indicated. The fold-stimulation (ratio of activity in the presence:absence of ligand) is indicated above each set of bars. The results are presented as means (± SD) from three independent experiments with each data point measured in triplicate (*n* = 3) where *ns* p≥0.05, ** p = 0.001–0.01, **** p<0.0001.

Given that the data depicted in Figs. 1, 2A and 2B were obtained with receptor constructs that encode the LBD of VDR, we then determined the impact of SENPs upon the transcriptional responsiveness of the full length version of this receptor assessed in the context of three different cell lines. In these experiments we employed: a Gal4-response element based reporter used in combination with hybrid constructs containing the full length VDR and; a reporter based upon the human *CYP24A1* gene promoter to assess the ability of SENPs to modulate VDRE-mediated activation by the VDR-RXRα heterodimer. Fig. 3 demonstrates that in HEK-293 cells, SENP1 and 2 were both able to enhance the vitamin D signal using both the Gal4 (A) and CYP24A1 (B) reporter systems. Immunoblot analysis of cellular lysates produced in Fig. 3A verifies that the increase in transcriptional potency is not a consequence of altered hybrid receptor protein levels (left middle panel) while both SENP proteins are noted to be expressed equivalently under our experimental conditions (right middle panel). When examined in the context of Caco-2 colon carcinoma cells, a similar profile of co-activation by SENP1 and SENP2 is achieved using the same Gal4 (C) and CYP24A1 (D) reporter systems. A general observation from experiments using the Gal4 system is that the impact of SENP upon receptor activation is even more effective when employing constructs expressing the full length VDR protein as compared to its LBD version. In contrast, Fig. 4 illustrates that the effects of SENP co-expression upon the 1,25D signal are remarkably diminished when assessed using MCF-7 breast cancer cells. In this cell model, neither SENP1 nor SENP2 has any effect on the transcriptional activity of VDR when examined using the Gal4 reporter (Fig. 4A) with similar effects observed with the CYP24A1-based system (Fig. 4B). In contrast, the RXRα-mediated signal was observed to be profoundly enhanced through SENP co-expression in these cells, with SENP1 eliciting the most potent effect upon activation of this receptor (Fig. 4A).

### Hormone-dependent Expression of an Endogenous Vitamin D Target Gene is Significantly Enhanced by SENP1

**Figure 5:**
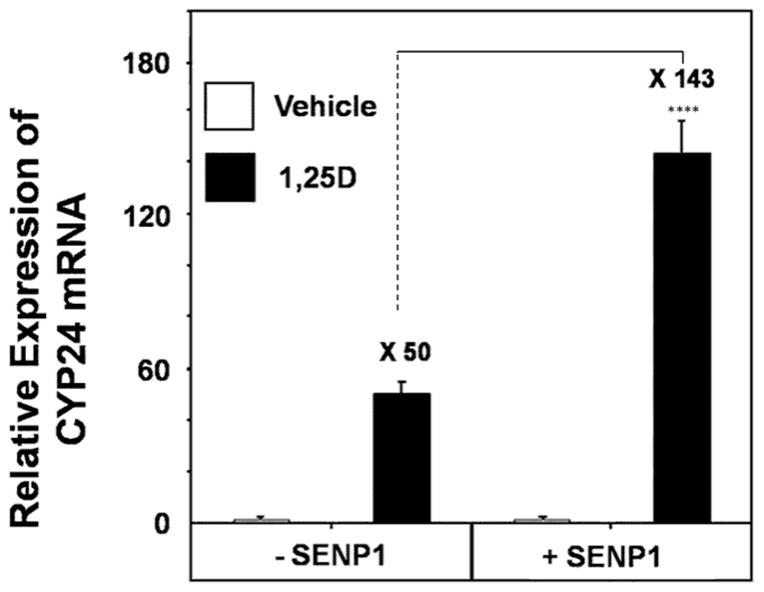
SENP1 potentiates the hormone responsiveness of an endogenous vitamin D target gene. Caco-2 cells were plated as described in methods and co-transfected with pSG5hVDR and, where indicated, pFLAG-CMV-SENP1 or corresponding parent vector control. Following incubation for a period of 24 hours with 1,25D (10 nM) or vehicle control, total RNA was isolated from cells, converted to cDNA and real time PCR analysis performed. The fold-stimulation of *CYP24A1* mRNA expression achieved through the presence of 1,25D is indicated above the black bars. The depicted data represents an average of three independent experiments with each data point a means (± SD) of triplicate assays (*n = *3) and **** p<0.0001.

To assess the effects of SENP1 upon an endogenous vitamin D target gene, we next employed real time PCR analysis to evaluate the impact of SENP1 over-expression within Caco-2 cells upon the transcriptional response of the *CYP24A1* gene to 1,25D. The data depicted in Fig. 5 demonstrate that exposure of Caco-2 cells to 1,25D (10 nM) for a 24 hour period will typically result in a 50-fold increase in the detectable levels of *CYP24A1* mRNA when compared to vehicle treated cells. Remarkably, for Caco-2 cells that prior received the SENP1 expression vector, the 1,25D-driven induction of *CYP24A1* mRNA became further increased to 143-fold, signifying an approximate increase of 300% in the hormone responsiveness of this vitamin D target through the addition of exogenous SENP1. Comparable results are observed for similarly-treated Caco-2 cells exposed to 1,25D for a 4 hour time period (data not shown).

### Knockdown of Endogenous SENP1 will Diminish the Ability of 1,25D to Induce CYP24A1 Gene Expression

**Figure 6:**
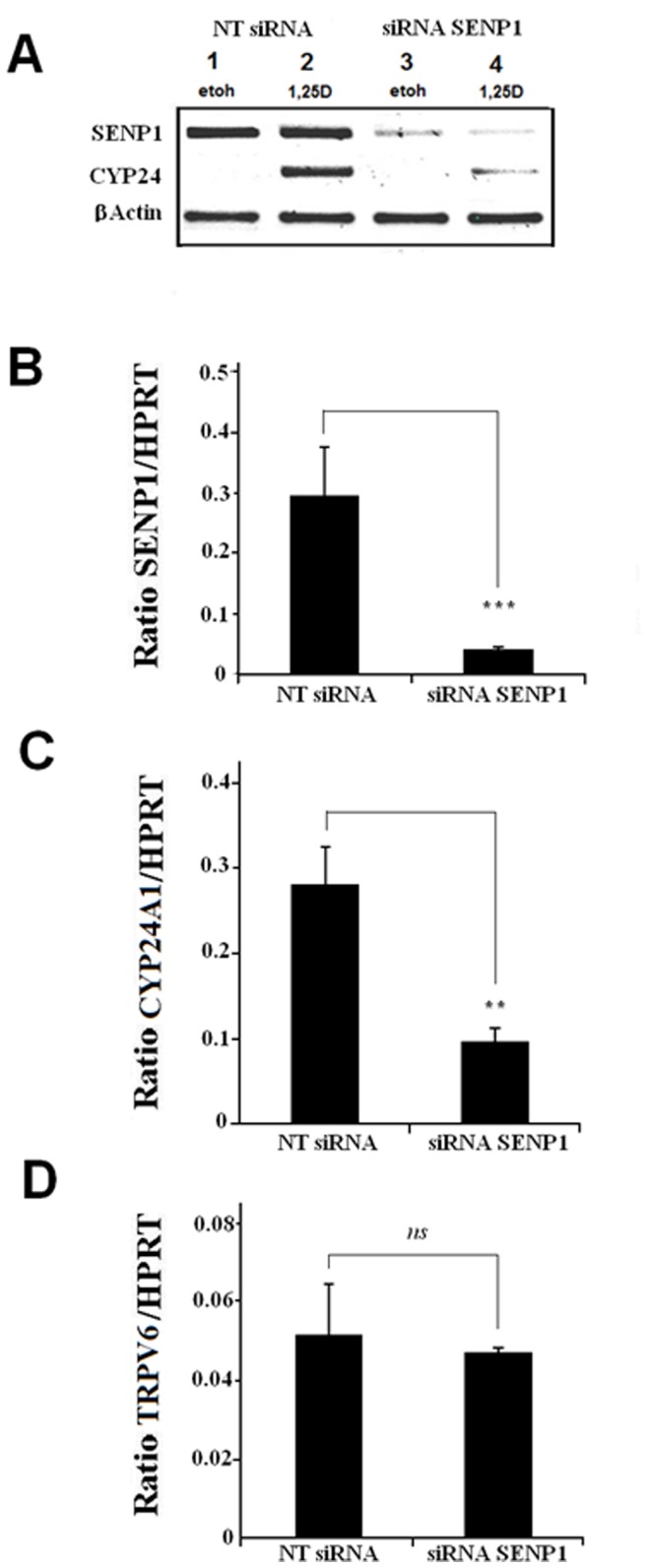
Depletion of endogenous SENP1 results in a diminished induction of *CYP24A* mRNA expression by 1,25D. Caco-2 cells were seeded and co-transfected with 100nM Dharmafect SENP1 siRNA or Non-Targeting (NT) control or as described in methods. Cells were then treated with vehicle or 1,25D (10^−8 ^M) for 24 hours before RNA/protein extraction and subsequent analysis through RT-PCR/qRT-PCR and immunoblotting. **A**. RT-PCR depicting gene specific PCR products obtained from Caco-2 cells transfected with non-targeting (NT) and SENP1-specific siRNAs and subsequently treated with 1,25D or vehicle control. The lower panels describe Q-PCR analysis of the impact of NT and SENP1-specific siRNAs upon the mRNA expression within 1,25D treated Caco-2 cells of (**B**) *SENP1*, (**C**) *CYP24A1* and (**D**) *TRPV6*. The experiment depicted in **A** is representative of three independent experiments while **B, C** and **D** describe the average of three independent experiments where each data point represents the means (± SD) of triplicate assays (*n = *3) and where *ns* p≥0.05, ** p = 0.001–0.01, **** p<0.0001.

As our previous experiments relate the impact of SENP1 overexpression, we next probed how the endogenous 1,25D response would be affected through depletion of cellular SENP1 through application of siRNA. Fig. 6A visually confirms that a gene-specific siRNA against *SENP1* will result in a reduced mRNA expression for this gene within Caco-2 cells with this effect maintained following exposure to 1,25D or vehicle control. Intriguingly when compared to the non-targeting (NT) control, a diminished transcriptional response of the *CYP24A1* gene to 1,25D is noted within SENP1 depleted cells (second row; compare lane 4 with lane 2), an effect not related to a lower level of VDR protein as confirmed through immunoblotting of corresponding lysate samples (data not shown). Q-PCR analysis confirms that the gene-specific siRNA will achieve a ‘knockdown’ of SENP1 mRNA expression in the 1,25D treatment group by approximately 80% (Fig. 6A) and a transcriptional response of *CYP24A1* that is diminished by approximately 65% (Fig. 6C). In contrast, depletion of cellular SENP1 has no statistically significant effect upon the mRNA expression of *TRPV6* (Fig. 6D).

### SENP1 and SENP2 Reverse Modification of VDR with SUMO2

**Figure 7:**
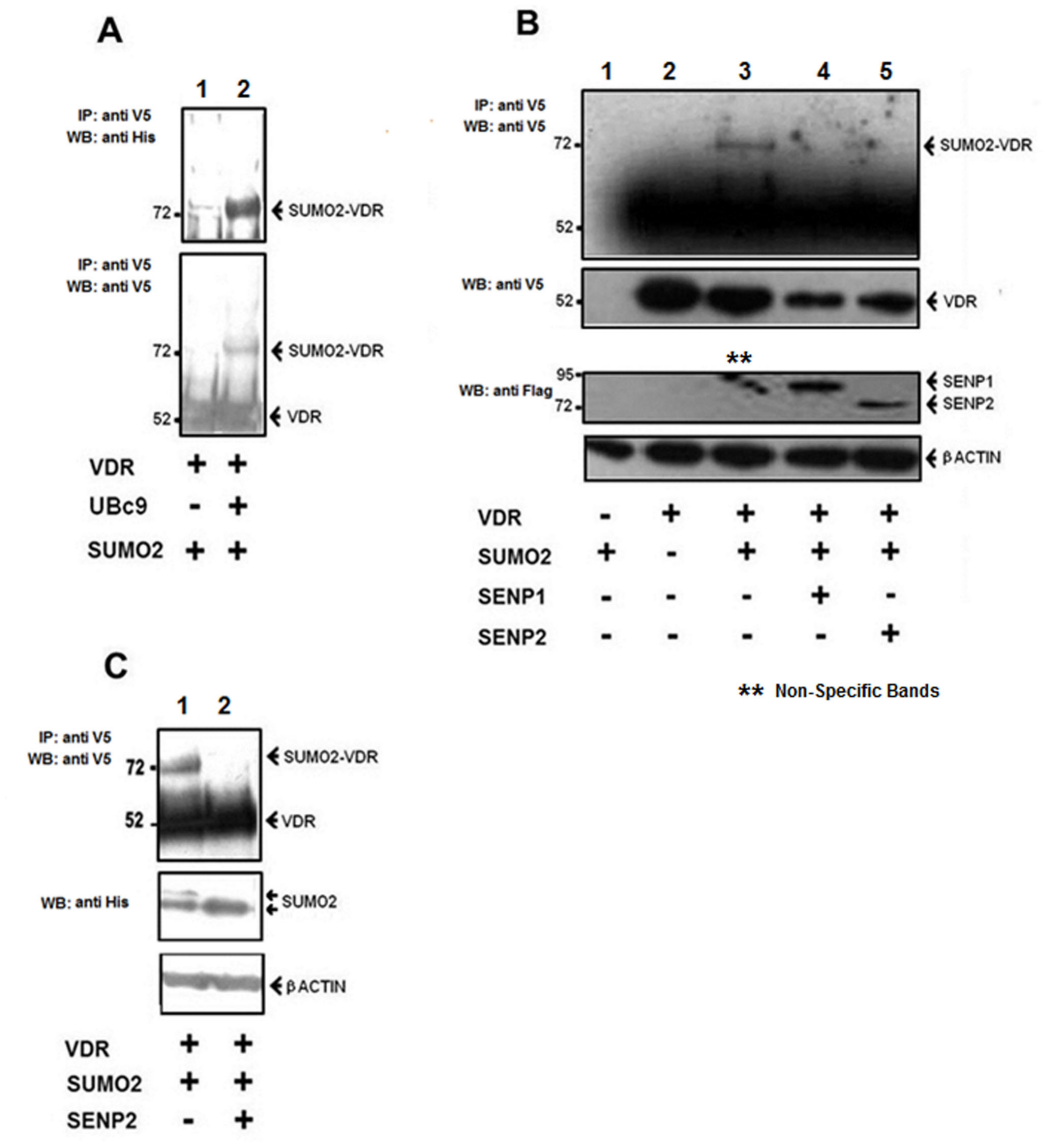
SENPs facilitate deSUMOylation of VDR. Depicted are cell-based SUMOylation assays performed as described in materials and methods using the following experimental conditions: A. HEK-293 cells received expression constructs for V5-VDR, His-SUMO2 and UBC9 or parent control. Cell lysates from each treatment group were incubated with V5-agarose beads and the resulting precipitated material subjected to western blot analysis with antibodies specific for 6x His (upper panel) or V5 (lower panel) epitope tags. The arrowheads indicate unconjugated and SUMO2-modified versions of V5-VDR. B. Where indicated, cells were co-transfected with V5-VDR, His-SUMO2, UBC-9 in combination with FLAG-SENP-1 or FLAG-SENP-2 with (-) denoting inclusion of appropriate parent vector control. The upper panel describes detection of SUMO2-conjugated VDR within precipitated lysates with expression of V5-VDR and Flag-SENP within the appropriate treatment groups confirmed in the lower panel. C. Cells received the indicated combination of V5-VDR, His-SUMO2, UBC-9 along and FLAG-SENP-2 or parent vector control. Upper panel depicts detection of SUMO2 conjugated VDR and reversal of this modification by SENP2. Lower panels confirm the expression status of unconjugated SUMO2.

As the functionality and SUMO-modification status of several nuclear receptors are known to be modulated through SENP-directed function, we performed a series of cell-based assays as depicted in Fig. 7 to evaluate the impact of SENP overexpression upon the SUMO status of VDR. For this purpose we utilized our previously described cell-based assay system in which V5-VDR is expressed in combination with components of the SUMO conjugation pathway (−/+ SENP) and the resulting lysates then subjected to immunoprecipitation using a V5-specific polyclonal antibody followed by western blot analysis for the detection of V5-VDR and its modified forms. In Fig. 7A, we verify our previous findings that VDR is modified by SUMO2 with this event dependent upon the co-expression of Ubc9 (compare lanes 1 and 2). The SUMOylated VDR is detected as a 72 KD band within precipitated cell lysates through use of antibodies specific for ‘tagged’ His-SUMO2 and V5-VDR. Formation of the SUMO2 conjugated VDR is again demonstrated in Fig. 7B (lane 3), however when this assay is performed to encompass either the Flag-SENP1 (lane 4) or Flag-SENP2 (lane 5) expression constructs the modified VDR is no longer detected. The lower panels confirm expression of V5-VDR and Flag-SENP within the corresponding treatment groups in which we note a slightly reduced V5-VDR protein expression within those lysates that include SENP (compare lanes 1&2 with lanes 3&4). Fig. 7C further confirms the deSUMOylation of VDR, in this case through SENP2 co-expression (compares lanes 1 and 2) with the middle panel verifying the co-expression of His-SUMO2 in this experiment. In lane 2 (middle panel) it is noted that the presence of SENP2 results in a loss of the upper band that represents the dimeric-SUMO2 species and a resulting predominance of the single (mono) SUMO2 form, verifying the enzymatic functionality of the expressed SENP2 protein.

### Lysine 91 of VDR is a Minor SUMO Acceptor Site

**Figure 8:**
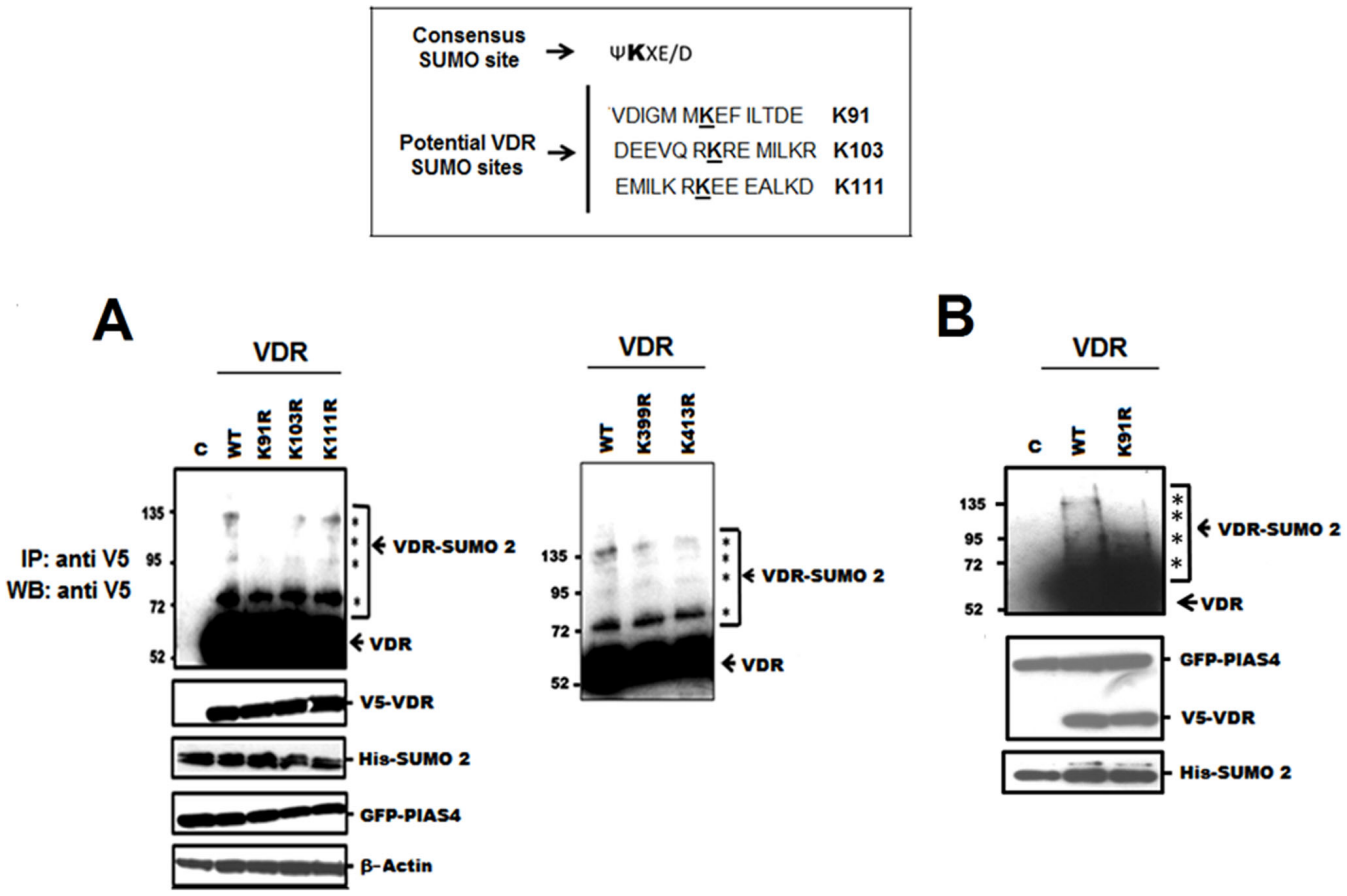
Identification of a possible SUMO acceptor site within VDR. Top: Predicted SUMO sites within VDR using the SUMOplot™ tool. A. Depicted are HEK-293 based SUMOylation assays of VDR mutant variants containing K to R substitutions at potential SUMO acceptor sites present within the C-terminal extension (left panel) and ubiquitin (K399R) and acetylation sites (middle panel). The asterisks denote SUMO2 modified VDR forms migrating at 72, 95 and 135 KD. B. SUMOylation assay of the wild type and K91R forms of VDR using HeLa cells that stably express SUMO2. All experiments included the expression construct for UBC9. Lower panels to both A and B confirm the equivalent expression of each mutant and individual components included in the assay.

In a preliminary attempt to identify which residue(s) within VDR may be subject to modification with SUMO, we employed site-directed mutagenesis analysis in which lysine at potential acceptor sites within VDR identified using the SUMOplot™ prediction tool (http://www.abgent.com.cn/doc/sumoplot/login.asp) was replaced with arginine. As detailed within Fig. 8, the three main potential conjugation sites (K91, K103 and K111) identified through inspection by this and other programs do not fulfill the ψΚxΕ criteria for a true consensus SUMO site. Each mutation was assessed through cell-based SUMOylation assays involving transient transfections of HEK-293 cells or Hela cells that stably express SUMO2. All experiments depicted in Fig. 8 included PIAS4, which we have previously reported to enhance SUMOylation of VDR in a fashion that may involve modification at more than one site [21]. Such a possibility is confirmed in Figure 8A in which inclusion of PIAS4 results in several SUMO-VDR species detected at 72, 95 and 132 KD. While none of the tested mutants exhibit a complete loss of receptor modification, we consistently observe that in the context of both the HEK-293 (Fig. 8A) and Hela (Fig. 8B) cell-based systems, the K91R mutant exhibits an overall reduced level of modification characterized by the disappearance of the SUMO-VDR species that migrates at 132 KD. These data intimate that residue K91 may represent a minor SUMO2 acceptor site. We also included in this analysis the K399R and K413R variants of VDR which represent potential ubiquitin [23] and acetylation [24] sites respectively, so as to determine if SUMO-modification of VDR was reliant on these processes. The data describe these mutants to possess a similar pattern of SUMO2 conjugation to that exhibited by wild type VDR (Figure 8A, right panel).

**Figure 9:**
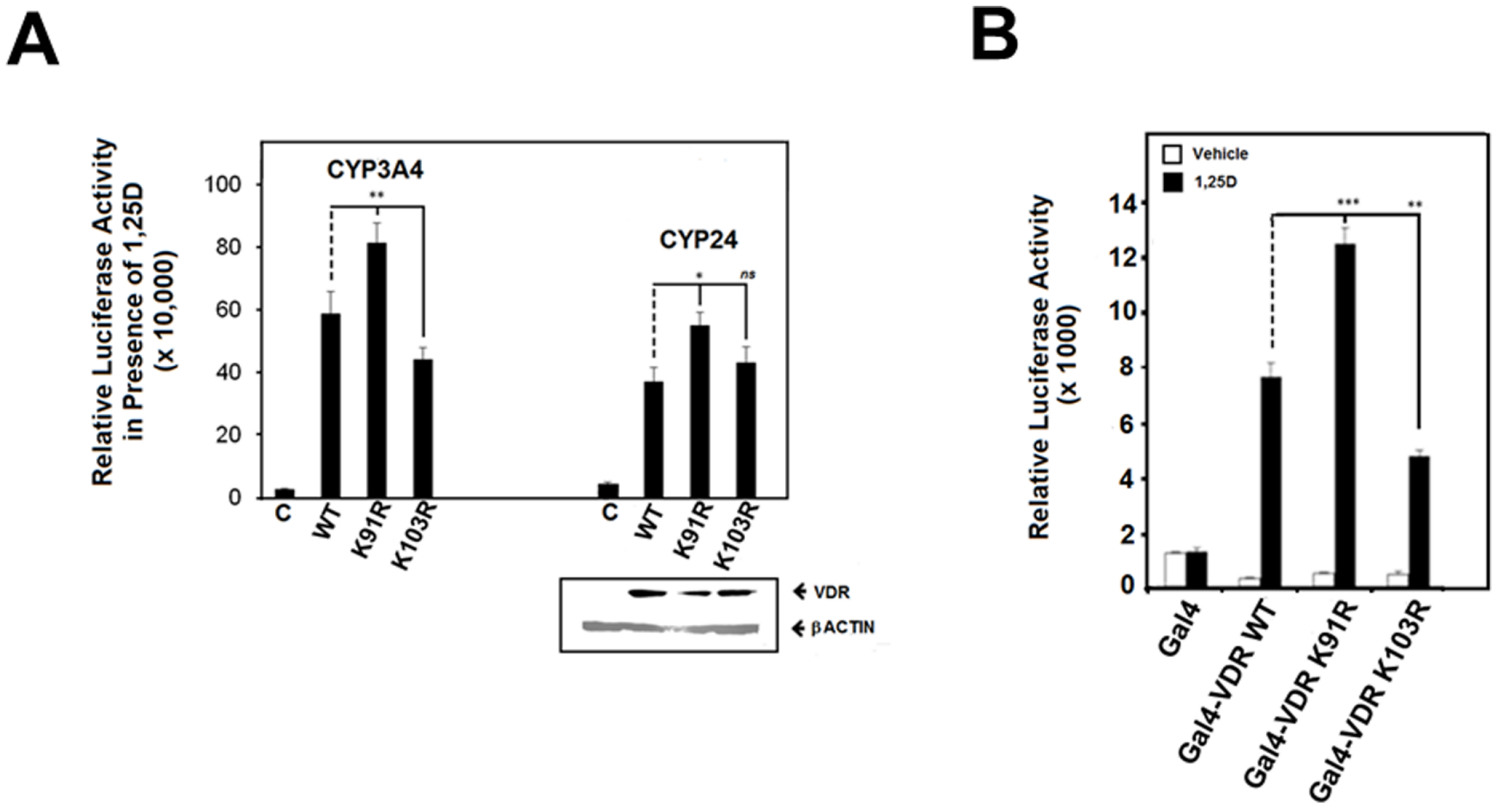
K91R VDR is associated with an increased level of transactivation by 1,25D. A. Functional activities of the wild type, K91R and K103R versions of VDR assessed using the pGI3-CYP3A4 and pMCS-24OHase luciferase-based reporter constructs. HEK-293 cells received the appropriate VDR and reporter construct in combination with expression vector for RXRα. The lower panel confirms protein expression of the wild type and mutant forms of VDR within same lysates that generate pMCS-24OHase reporter data. B. Transcriptional responses to ligand exhibited by the wild type, K91R and K103R forms of VDR expressed as Gal4 hybrid proteins and assessed through luciferase activity of the pFLUC reporter vector that contains five copies of the Gal4 response element. Cells were exposed to 1,25D for 24 hours before recording of luciferase values. All data within each figure represents means (± SE) of triplicate assays (*n* = 3) where *ns* p≥0.05, ** p = 0.001–0.01, *** p = 0.0001–0.001.

We then assessed transactivation of the K91R mutant by 1,25D in comparison to the wild type and K103R forms of VDR. Fig. 9 describes data obtained using the (A) CYP3A4 and CYP24A1 promoter constructs and the (B) Gal4-based reporter system. In each context, we consistently observe the K91R mutant to exhibit a higher level of transactivation compared to the intact VDR. This is most apparent when employing the Gal4 system in which the liganded activity of the K91R mutant is approximately 55% greater than that of the wild type VDR, while in contrast the responses of K103R VDR to 1,25D appear to be marginally diminished when assessed in the context of the CYP3A4 and Gal4 systems.

## Discussion

In this report we expand upon our previous findings that SUMO-related activity can modulate the vitamin D hormonal response and demonstrate that members of the SENP family can interact with VDR to reverse its modification with SUMO2 in addition to significantly potentiating its transactivation by the 1,25D ligand. The current study primarily focused on those effects exerted by SENP1 and SENP2 as their isopeptidase activities exhibit equal preference towards nuclear substrates modified with any of the three SUMO isoforms [9], [25]. Our experiments excluded SENP3 and SENP5 as they are located principally within the nucleolus and therefore their potential for interaction with VDR likely to occur under specific biological conditions outside the scope of this study [9], [25]. We do acknowledge that a complete appreciation of how SENPs impact upon the vitamin D response need in future also consider the activities of SENP6 and SENP7, which although are more modestly engaged in deconjugation of mono-SUMOylated proteins are localized within the nucleoplasm and possess a specialized capacity to dissemble poly SUMO2/3 chains [25]. This latter feature may be of relevance to VDR which we have demonstrated may exist as a multi or poly-SUMO2 conjugated protein as a result of the E3-ligase activities of PIAS4 [21]. In the context of our initial screening assay we find SENP1 to be a robust modulator of the ligand-induced transactivation of both VDR and its RXRα heterodimeric partner. That no comparable effects were observed for the PPARγ and LXRα constructs verifies our observations to be specific and not an inherent feature of the Gal4-based system. SUMOylation of the ligand binding domains of PPARγ and LXRα has been shown to be pivotal to their transrepression function [14], [16] and therefore SENP1 co-expression may not impact upon their transactivation by ligand when assessed through this assay. We confirm the effects of SENP1 using gene-reporter constructs and also in the context of the endogenous *CYP24A1* vitamin D target gene as evidenced through its profoundly enhanced response to hormone following SENP1 over-expression. Conversely, depletion of cellular SENP1 significantly diminished mRNA expression of *CYP24A1* but had little effect upon *TRPV6.* Such differing responses of these vitamin D target genes may be reflective of distinct SUMO-modification patterns for VDR and/or its co-regulatory complexes associated with differing chromatin contexts and warrant future consideration that SENP-mediated modulation of the vitamin D response may be gene-specific. Indeed, if SENPs do exert their most potent effects upon regulation of *CYP24A1*, then their enhancement of the expression of this gene and its associated catabolism of the 1,25D ligand may have implications as to SENP involvement in vitamin D resistance. SENP1 expression levels have been demonstrated to be elevated during prostate pathogenesis [26]–[27] while those of *CYP24A1* are increased in cancers of the prostate, colon, ovary, and lung, a number of which are known to be insensitive to the growth-regulatory effects of vitamin D [28], [29]. It is also important to consider that SENP1 and SENP2 may exhibit a degree of functional redundancy and so determination of the true extent of their influence upon the VDR-mediated signaling will warrant simultaneous knockdown of more than one SENP family member.

Our previous work has identified that the modification of VDR with SUMO2 is significantly enhanced through the E3-ligase activity of PIAS4 which also serves to repress the 1,25D transcriptional signal [21]. When taken in consideration with the current data, we propose a working model in which the principal effect of VDR SUMOylation is to increase the receptor population bound to co-repressor in a manner similar to the reported recruitment of NCoR1 by the SUMO1-conjugated form of PPARα [30]. Within the context of the VDR-RXR heterodimeric complex, binding of ligand would then result in conformational changes that facilitate recruitment of SENP(s) whose associated activities result in the release of co-repressors and/or the assembly of co-activator complexes. While the hormone-dependent interactions exhibited in our mammalian two hybrid system support such a role for SENPs, our pull-down data do not clearly indicate an influence of 1,25D upon formation of the VDR-SENP complex. It is possible that when assessed within the context of an intact cellular milieu, such as that represented by the two hybrid system, binding of 1,25D by VDR may lead to the recruitment of other proteins which help further stabilize interactions between SENP and receptor that are not achieved in the pull-down system. Such a scenario would suggest that an interacting SENP may also form contacts with one or more components associated with the liganded VDR or indeed the RXR heteropartner. Indeed, while we demonstrate that SENPs can deconjugate the SUMO2-modified VDR, it is conceivable that their ability to enhance the 1,25D transcriptional response may also be partially attributable to the deSUMOylation of other proteins within VDR-recruited proteins in a fashion similar to the enhanced responsiveness of AR to ligand that results from targeting of HDAC1 by SENP1 [31]. Such a mechanism involving VDR-associated proteins is supported by the apparent cell-line specific effects noted in this study in which SENP co-expression has little impact upon the vitamin D signal when assessed in MCF-7 breast cancer cells while under the same experimental conditions the RXRα transcriptional response is profoundly enhanced. Such findings may again reflect distinct cellular/tissue specific profiles of SUMO-substrates among VDR co-regulator proteins and/or varied patterns of SUMOylation associated with the receptor itself. This is an intriguing scenario that may provide a critical contributory mechanism underlying the pleiotropic effects of vitamin D and the variable sensitivity to VDR ligands exhibited between different cell types or disease states based upon altering SUMOylation of receptor/accessory proteins. In addition, our previous report observed that over-expression of PIAS4, in addition to enhancing SUMOylation of VDR, also increased the protein levels of this receptor [21]. In this current study we note that co-expression of SENP1 or SENP2 is associated with a reduced level of VDR protein and so it will be intriguing to establish if a functional link exists between the SUMOylation status of VDR and receptor stability/degradation. It is possible that SUMOylation may inhibit the VDR-mediated signal through impeding receptor clearance from the promoters of target genes at the later stages of the VDR transcriptional ‘life cycle’ [32] with this then reversed through SENP-directed activity.

*In silico* screening reveals that while VDR lacks a true SUMO consensus sequence, this receptor does possess a number of potential type II (non-consensus) acceptor sites that include K91, K103 and K111, all located within a region previously defined as a C-terminal extension (CTE) of the core zinc finger DNA binding domain [33]. While our initial analysis reveal none of these residues represent the predominant contributor to VDR SUMOylation, our data do describe K91 as a minor acceptor site as implicated though the loss of the higher migrating SUMO-VDR species that typically form in the presence of PIAS4. K91, along with E92 are components of the T-box located within the CTE of VDR and are critical for mediating dimerization with RXR and binding of the heterocomplex to DNA [34], [35]. A previous report described replacement of K91 and E92 with asparagine and glutamine respectively that result in an almost complete reduction in transcriptional activity [35]. Our present study details that the more conservative replacement of K91 with arginine will avoid such a dramatic loss of activity, likely due to the mutant variant retaining the capacity to form salt bridges with the D-box of RXR. The loss of SUMOylation that accompanies the K91R change is accompanied by an increased level of VDR transactivation in response to cognate ligand, thus supporting our hypothesis that this post-translational modification will exert a repressive function upon VDR. In the specific case of K91, such effects of SUMOylation are possibly mediated through impeding formation of the VDR-RXR heterodimer and its association with DNA. In contrast, in substituting K103 with arginine we generated a VDR variant that exhibits a modest decrease in ligand responsiveness, particularly when assessed in the context of the CYP3A4 promoter and Gal4 reporter systems. These findings correlate with the findings of Hsieh and co-workers in which the K103A and K111A mutant forms of VDR exhibited an even greater loss in transcriptional potency, reflecting their potential role as contact sites for transcriptional co-activators or basal transcription factors [33]. It is an on-going program of research in our laboratory to probe VDR for the remaining cryptic SUMO acceptor sites utilizing additional methodologies to creating single site mutants.

Throughout this study we also consistently observed that SENP1 potently enhanced the ligand-induced activation of RXRα. It has been reported that K108, located within the AF-1 region of RXRα, is an acceptor site for SUMO1 and that reversal of this modification by SUSP1 (SENP6) leads to an enhanced receptor activity [36]. The same study also observed that SENP1 was unable to deconjugate the SUMO1-modified RXRα and had no detectable effect upon its transcriptional activation. While we also note enhancement of RXRα activity by SENP6 to levels comparable to that reported by Choi and co-workers (data not shown), we consistently find that its potentiating effects are modest in comparison to those elicited through SENP1 when evaluated in the context of the Gal4 system and also RXRE-based reporter assays which employ the intact native form of RXRα (data not shown). We are uncertain of the exact reason(s) that underlie the discrepancy between our findings with respect to the actions of SENP1 on RXRα, particularly when our two studies have employed similar methodologies and cell types. It should also be noted that the RXRα LBD construct utilized in our initial screen (Fig. 1) encompasses residues 197 to 462 and so excludes K108, but still exhibits a profound capacity to be stimulated by SENP1. These data suggest that RXRα may contain SUMO acceptor sites that are additional to K108, although an alternative explanation is again that SENP1 may be targeting deSUMOylation of an RXRα protein co-factor.

In total, our novel data reveal that the process of reversible SUMOylation to represent a mechanism that may enable ‘fine-tuning’ of the transcriptional activities of the VDR/RXRα heterodimer in a cell and gene specific context and involve modification of VDR at one than one site. Additional characterization of this process and its modulation with respect to VDR and RXR in addition to those protein co-factors recruited to this heterodimeric complex will be critical to understanding the molecular basis that underlies the cell, tissue and promoter-specific responses to the endocrine and dietary-derived ligands of VDR.
